# Insights Into the Biological Role of NEDD4L E3 Ubiquitin Ligase in Human Cancers

**DOI:** 10.3389/fonc.2021.774648

**Published:** 2021-11-15

**Authors:** Shangdan Xie, Lu Xia, Yizuo Song, Hejing Liu, Zhi-wei Wang, Xueqiong Zhu

**Affiliations:** Department of Obstetrics and Gynecology, The Second Affiliated Hospital of Wenzhou Medical University, Wenzhou, China

**Keywords:** NEDD4L, ubiquitination, cancer, treatment, degradation

## Abstract

Neural precursor cell expressed developmentally downregulated 4-like (NEDD4L) is an E3 ubiquitin ligase that has been reported to participate in multiple cellular procedures by regulating of substrate ubiquitination and subsequent protein degradation. A great amount of evidence has demonstrated that NEDD4L mainly functions as a tumor suppressor in most cancer types, while it also acts as an oncogene in a few cancers. In this review, we summarize the potential role of NEDD4L in carcinogenesis and the related underlying molecular mechanism to improve our understanding of its functions in the tumorigenesis of human malignancies. Developing clinical drugs targeting NEDD4L could be a potential therapeutic strategy for cancer therapy in the future.

## Introduction

Ubiquitination is a cellular biological process that specifically modifies posttranslational proteins and results in substrate degradation, stabilization or relocation (1). Several successive enzyme reactions constitute a cascade of ubiquitination. Ubiquitin was originally linked to E1 ubiquitin activating enzymes for its activation in an ATP-dependent manner and then instantaneously shifted to E2 ubiquitin conjugating enzymes. Afterward, the transfer of ubiquitin from E2 to the substrate is mediated by E3 ubiquitin protein ligases (E3s) (2, 3). Abnormalities of ubiquitination have been confirmed to be related to tumor occurrence and development (4). It is known that E3s determine the substrate specificity of the system to a large extent (5); therefore, the disorders of E3s might lead to the initiation of human cancer.

To date, there have been more than 600 E3s in the human genome, which are mainly sorted into three categories: really interesting new gene (RING) finger family E3s, the RING-between-RING (RBR) family E3s, and homologous to the E6-AP C-terminus (HECT) family E3s (6). HECT family E3s have an active site for cysteine, where cysteine binds to ubiquitin for an intervening thioester prior to its substrate (7). Specific recognition of substrates is based on the nonconservative N-terminus of HECT family E3s (8). As the most familiar and studied group of HECT family E3s, the neural precursor cell expressed developmentally downregulated 4 (NEDD4) subfamily has nine members, including NEDD4 (known as NEDD4-1), NEDD4-like (NEDD4L, also named NEDD4-2), ITCH, Smurf1, Smurf2, WWP1, WWP2, NEDL1 (also named HECW1) and NEDL2 (also named HECW2), and is characterized by a WW domain and a C2 domain (9, 10). NEDD4-like, is a member of the NEDD4 subfamily and is reported to regulate various ion channels and virus budding (11). NEDD4L plays a potential role in the growth of the central nervous system, the regulation of hypertension and the development of cancer and so on (12). In recent years, compelling evidence has shown that NEDD4L accelerates or weakens the progression of various types of cancers by targeting different substrates (13, 14). As a result, this review presents the structure of NEDD4L and aims to summarize the function of NEDD4L in diverse cancer types.

## Structure and Function of NEDD4L

**Figure 1:**
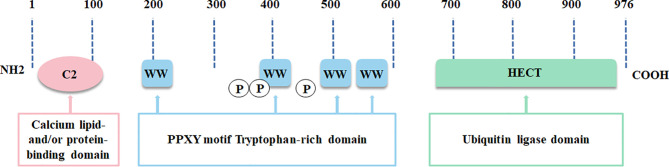
The structure of NEDD4L is illustrated.

In the NEDD4 ubiquitin ligase family, NEDD4L is the most analogous homolog of NEDD4, the archetypal member of the family (15). NEDD4L is widely distributed and highly conserved in vertebrates (11). Human NEDD4L is located on chromosome 18q21.31 and has 41 exons. NEDD4L exists as two protein bands in various tissues, including human tissue, one of which changes marginally due to tissue specificity, and the other one, a stably expressed protein, containing an N-terminal C2 domain, 4 WW domains and a C-terminal HECT domain (**Figure 1**) (11). The chief function of the C2 domain consists of Ca^2+^ binding, membrane targeting and protein-protein interactions (16). The WW domains play a pivotal role in discriminating and provoking the specific substrates of NEDD4L (17). The HECT domain contains the catalytic cysteine 42 and participates in catalyzing polyubiquitin chain packaging, a two-step mechanism implicated in two E2 ubiquitin binding sites (18). The C2 domain and the HECT domain restrain each other to control their own activity in normal circumstances (19). This state of equilibrium is broken when intracellular Ca^2+^ binds to the C2 domain, thus stimulating the ubiquitin ligase activity of NEDD4L and then recruiting it to the plasma membrane (20). Expectedly assembling substrate-linked ubiquitin chains, including Lys-63, Lys-48, Lys-27, Lys-11 and Lys-6 linkages, NEDD4L can result in substrate degradation by lysosomes or the proteasome, and/or change the cell signaling pathway (18, 21, 22).

The original and most familiar function of NEDD4L is to regulate the epithelial Na^+^ channel (ENaC), which is closely related to the fluctuation of blood pressure (23). In addition, NEDD4L also participates in DNA repair, antiviral immunity and tumor development (24–26). To date, multiple reports have shown that the expression of NEDD4L in cancers is abnormal, and various proteins have been validated to bind with NEDD4L or be ubiquitinated by NEDD4L, thus modulating the cancer development (27). For instance, DNA damage-binding protein 2 (DDB2) suppresses the expression of NEDD4L and then affects the transforming growth factor-β (TGF-β) signaling in ovarian cancer (28). This review focuses on the potential role and related molecular mechanisms of NEDD4L in carcinogenesis and tumor progression.

## The Involvement of NEDD4L in Different Signaling Pathways

NEDD4L is implicated in the regulation of various signaling pathways. For example, NEDD4L induced the ubiquitination of Unc51-like kinase 1 (ULK1), an autophagy initiation related protein, to control autophagy (29, 30). Endoplasmic reticulum stress increases the level of NEDD4L to trigger autophagy (31). 8-Oxoguanine DNA glycosylase (OGG1) is the central cellular enzyme applied in the excision of 8-oxoguanine DNA base lesions in DNA *via* the base excision repair pathway, and NEDD4L is involved in OGG1 ubiquitylation in response to DNA damage (24). In addition, NEDD4L catalyzed Dishevelled 2 (Dvl2) polyubiquitination, and Dvl2 was considered as a major mediator of both Wnt/β-catenin and Wnt/planar cell polarity pathways (21), suggesting that NEDD4L inhibited Wnt signaling. In addition, NEDD4L mediated the ubiquitination of PIK3CA and then weakened PI3K-AKT signaling (32). It was also reported that NEDD4L might enhance MAPK/ERK signaling but few studies have focused on concrete mechanisms (13). The specific recognition of the TGF-β-triggered phosphoThr-ProTyr motif in the junction region by the WW domain of NEDD4L contributed to Smad2/3 polyubiquitination and degradation, thus inhibiting TGF-β signaling (33).

## The Function of NEDD4L in Cancer

### Gastric Cancer

Gastric cancer ranks the fifth among the most common cancers and is the third-leading cause of cancer death (34). To date, it has been reported that the expression of NEDD4L is positively related to the outcomes of gastric cancer patients (35). Patients with negative NEDD4L expression tended to have lymphatic infiltration, metastasis and vascular invasion in sharp contrast to those with positive NEDD4L expression (35). In addition, a high level of NEDD4L generally corresponded to a low level of HIF-1α in gastric cancer tissues and led to a favorable prognosis (36). Collectively, NEDD4L might affect the metastasis of gastric cancer and together with HIF-1α, could predict the prognosis of patients. However, further experiments at the cellular and animal levels are needed to test the function of NEDD4L in gastric cancer development.

### Liver Cancer

Liver cancer, as the second most lethal cancer worldwide, emerges in approximately 900, 000 people and causes about 830, 000 patient deaths every year (37). In hepatocellular carcinoma (HCC), the Wnt/β-catenin signaling pathway is frequently abnormally regulated (38). A study showed that the expression of NEDD4L was elevated when Wnt/β-catenin was activated in HCC (39). However, another study revealed that the decreased expression of NEDD4L could enhance the proliferation ability of HCC cells (13). In an *in vivo* experiment, overexpression of NEDD4L attenuated the growth of xenograft tumors in nude mice (13). In addition, both of *in vivo* and *in vitro* experiments suggested that NEDD4L might advance the MAPK/ERK signaling pathway to weaken the proliferation of HCC cells *via* the induction of apoptosis (13). In patients, it was found that the expression of NEDD4L in HCC tissues was lower than that in paracancerous tissues and the patients with high NEDD4L expression had better outcomes than those with low expression of NEDD4L (13). Consequently, NEDD4L might act as a tumor-suppressor gene to repress the malignant biological behavior of HCC, while the relationship between NEDD4L and the Wnt/β-catenin signaling pathway needs further investigation to prove.

### Colorectal Cancer

Colorectal cancer (CRC) is the third most frequent cancer and the second most deadly cancer globally (37). By inhibiting the destruction of the LGR5 receptor, the absence of NEDD4L could facilitate the signal transduction of Wnt/β-catenin and the number of intestinal stem cells, thus promoting the susceptibility and progression of colorectal tumors (40). Moreover, the expression of NEDD4L was all reduced in various stages of colorectal cancer specimens, in contrast to that in adjacent normal mucosal tissues. NEDD4L significantly suppressed the Wnt/β-catenin signaling pathway in CRC cells (41). NEDD4L can degrade serine/threonine kinase 35 (STK35) by ubiquitination and then inhibit glycolysis, increasing the apoptosis of CRC cells by suppressing the Akt signaling pathway and modulating the chemoresistance of CRC cells (42). In addition, a study revealed that the expression of NEDD4L in rectal cancer patients was upregulated after radiotherapy, suggesting that NEDD4L might be helpful for the treatment of rectal cancer patients (43). Taken together, NEDD4L possibly weakens the development of CRC and more *in vivo* experiments are required to validate the function of NEDD4L.

### Pancreatic Cancer

Pancreatic cancer is a malignant cancer with a poor prognosis and takes approximately 466,000 lives away every year (37). Mounting studies have discovered that NEDD4L plays a pivotal role in the development of pancreatic cancer. MicroRNA-23A (miR-23A) inhibits the expression of NEDD4L to collaborate with miR-21 and miR-27A, thus promoting the progression of pancreatic cancer (44, 45). NEDD4L was demonstrated to be linked to iron metabolism in pancreatic cancer. N-myc downstream regulated gene-1 (NDRG1), an iron-regulated metastasis suppressor, upregulated the expression of NEDD4L in PANC1 pancreatic cancer cells (46). NEDD4L mediates the degradation of the iron-binding transport protein lactotransferrin (LTF) by ubiquitination to hinder the malignant biological behavior of pancreatic cancer (47). Besides, NEDD4L impeded autophagy and cancer cell proliferation by being involved in the degradation of ULK1, and weakening the expression of the glutamine transporter ASCT2 by the ubiquitination pathway in pancreatic cancer (30). Hence, NEDD4L acts as a tumor-suppressor gene in pancreatic cancer.

### Gallbladder Cancer

Although gallbladder cancer is an uncommon cancer, delayed diagnosis and poor prognosis are distinctive features (48). A study demonstrated that the expression of NEDD4L in the cytoplasm of invasive cancer cells is much higher than that in normal or dysplastic epithelial cells (14). Furthermore, NEDD4L enhanced the invasion ability of gallbladder carcinoma cells by increasing metalloproteinase-1 (MMP-1) and MMP-13 expression (14). Interestingly, downregulation of NEDD4L did not affect cell growth in gallbladder cancer (14). Unlike with most cancer types, NEDD4L might exert a tumor-promoting effect on gallbladder cancer.

### Lung Cancer

Lung cancer is the main cause of cancer death and seriously threatens the lives of people worldwide (49). It was reported that NEDD4L is considered as one of the central drivers in lung adenocarcinoma (LUAD) and might affect the prognosis of the patients (50). Downregulation of NEDD4L was found in nonsmall cell lung cancer (NSCLC) samples in contrast to those of normal tissues. In NSCLC patients, NEDD4L rs11660748 A>G and rs73440898 A>G had adjusted hazard ratios (HRs) of 1.31 and 1.27, respectively, for overall survival, which means that the mutations at these two sites might impair the prognosis of patients. Moreover, low expression of NEDD4L was prone to lymph node invasion, late stage and deprived prognosis (51, 52). These results suggest that NEDD4L might inhibit the progression of lung cancer. Further studies demonstrated that NEDD4L suppressed the proliferation, migration and invasion of lung cancer cells (51). Enhancer of zeste homolog 2 (EZH2) is a highly conserved histone methyltransferase (HMTase) and is abnormally expressed in various cancers (53). Notably, EZH2 could weaken the expression of NEDD4L to serve as an oncogene in lung cancer (51). In addition, miR-93 is a member of the miR-106b-25 cluster and a driving force for the development of numerous cancers, such as bladder cancer, prostate cancer and lung cancer (54–56). MiR-93 mediated the decrease in NEDD4L expression and then enhanced TGF-β signaling to activate epithelial-to-mesenchymal transition (EMT) in lung cancer (57). Tumor-associated macrophages (TAMs) play a pivotal role in tumor aggressiveness and M2 macrophage-derived exosomes (MDEs) are critical communication media in the tumor microenvironment (50). MDE was reported to reduce the expression of NEDD4L and then stabilize the c-Myc protein to induce chemoresistance in lung cancer by transferring miR-3679-5p to cancer cells (58). In addition, NEDD4L be involved in the ubiquitination of multidrug resistance-associated protein 1 (MRP1), which was negatively correlated with the prognosis of patients with lung cancer (59). However, NEDD4L might act as an oncogene to some extent. NEDD4L could repress the expression of general control nonderepressible kinase 2 (GCN2) to control its proapoptotic effect on lung cancer cells (60). Overexpression of GCN2 aggravated cell apoptosis that was induced by Na^+^, K^+^-ATPase ligand in A549 lung cancer cells, indicating that NEDD4 might repress apoptosis of lung cancer cells by targeting GCN2 (60). In summary, NEDD4L could inhibit the progression of lung cancer by targeting multiple signaling pathways, but might suppress the apoptosis of cancer cells to a certain extent.

### Nasopharyngeal Carcinoma

Nasopharyngeal carcinoma is an epithelial carcinoma that is closely related to the Epstein -Barr virus (EBV) infection. There are approximately 133,000 new cases and 80,000 deaths worldwide every year (37). A study utilized whole-exome capture/sequencing in 251 patients with different EBV infections (205 affected, 21 obligate carriers and 25 unaffected) and revealed that NEDD4L might regulate EBV infection (61). However, the mechanism of modulating EBV infection by NEDD4L and the role of NEDD4L in the initiation and progression of nasopharyngeal carcinoma are poorly understood. Further studies are needed to verify the function of NEDD4L in nasopharyngeal carcinoma.

### Ovarian Cancer

Ovarian cancer is one of the top 10 most common cancers in females, with an approximately 46% five-year survival rate (62). The expression of NEDD4L was reduced in invasive ovarian cancer tissues in sharp contrast to that in normal ovarian epithelial tissues (63). Furthermore, the patients with higher levels of NEDD4L tended to have an early clinical stage, few lymph node metastases and good survival (63). However, NEDD4L might exert a tumor-promoting effect, and the expression of NEDD4L was downregulated by DNA damage-binding protein 2 (DDB2) (28). DDB2 participates in many biological processes including gene transcription and cell cycle regulation and is identified as a critical factor in tumor development (64, 65). DDB2 could attenuate the expression of NEDD4L and then stimulate TGF-β signaling to inhibit the proliferation of ovarian cancer cells (28). These data suggested that TGF-β might be a potential substrate of NEDD4L. However, the mechanism of inhibition of NEDD4L on TGF-β signaling was not investigated in this study; thus, more studies are needed to verify the relationship between NEDD4L and TGF-β. Few studies have investigated the independent effect of NEDD4L on ovarian cancer, and more research needs to be carried out.

### Endometrial Cancer

Endometrial cancer is the sixth most common cancer in women, and the incidence and mortality of this cancer have increased in recent years (59). Endometrial cancer tissues showed decreased expression of NED44L compared with endometrial hyperplasia tissues utilizing immunohistochemical staining (66). However, there is a lack of research investigating the relationship between the level of NEDD4L and the initiation, development and outcomes of endometrial cancer.

### Prostate Cancer

The expression and functions of NEDD4L in prostate cancer are still ambiguous. The expression of NEDD4L was decreased in prostate cancer specimens compared with benign prostatic hyperplasia (67). Interestingly, the expression of three NEDD4L transcripts, NEDD4Lf, NEDD4Lg and NEDD4Lh, was upregulated in prostate cancer cells after androgen administration (68, 69). NEDD4L was also downregulated in androgen-independent prostate cancer cells (70). It was suggested that the dysregulation of NEDD4L might result from the level of androgen. Additionally, SOX5 and DNA methylation possibly acted as regulators of NEDD4L in androgen-independent cancer cells (70). However, the study failed to show the molecular mechanisms by which NEDD4L modulated prostate carcinogenesis. Unexpectedly, Hellwinkel et al. reported that the level of NEDD4L was higher in prostate cancer tissues than in adjacent normal tissues (71). Furthermore, NEDD4L might contribute to the development of prostate cancer by reducing the TGF-β signaling pathway (71). Hence, the function of NEDD4L in prostate cancer is not clear.

### Renal Cancer

Renal cell carcinoma (RCC) is a common malignant tumor in the urinary system (37). It was reported that the expression of NEDD4L was positively related to overall survival and disease-specific survival (DSS) in clear cell renal cell carcinoma (ccRCC) and chromophobe cell renal carcinoma (CCRC) (72, 73). In further studies, NEDD4L could limit the proliferation and metastasis of ccRCC cells by weakening the ERBB3 and MAPK signaling pathways (73). Consequently, NEDD4L can be considered as a potential therapeutic target in ccRCC.

### Breast Cancer

Breast cancer is the most common malignancy in females, and hormones, reproduction and lifestyle may affect the tumorigenesis of breast cancer (74). It was reported that a high level of NEDD4L might indicate a beneficial prognosis with free recurrence (75). In various breast cancer cells, miR-106b-25 could inhibit the expression of NEDD4L to upregulate the level of NOTCH1, which was instrumental in tumor-initiating cell (TIC) induction (75, 76). In addition, pseudokinase Tribble 3 (TRIB3) interacted with Akt and repressed NEDD4L-mediated ubiquitination of Forkhead box O1 (FOXO1) and then promoted the expression of Sry-related high-mobility box 2 (SOX2), a transcription factor of cancer stem cells, to exacerbate the development of breast cancer stem cells (77). NEDD4L modulated the degradation of copper transporter 1 (CTR1) in ubiquitination and exerted tumor inhibition through the CTR1-Akt signaling pathway (78). Several studies found that NEDD4L was probably implicated in the anticancer or cancer-promoting effects of some substances in breast cancer. For instance, selenium (Se) is a lurking anticancer nutrient (79), and NEDD4L is considered as a key gene in the regulatory network of the Se-stimulated epigenome (80). Unexpectedly, Orai3 advanced calcium influx, activated NEDD4L and reduced the apoptosis of breast cancer cells by weakening the expression of p53 (81). The relationship between NEDD4L and p53 has been reported, and NEDD4L might promote the survival of cancer cells (82). In brief, most research suggests that NEDD4L suppresses the malignant biological behavior of breast cancer cells, and further studies are necessary to discover its underlying mechanism in breast carcinogenesis.

### Other Human Cancers

NEDD4L, activated by a functional polypeptide (JP1), induced degradation of SP1 *via* the ubiquitin-proteasome pathway and then attenuated the transcription of integrin αvβ3 to inhibit cell proliferation and metastasis of melanoma (83). In addition, serum- and glucocorticoid-regulated kinase 1 (SGK1) inhibited the degradation of the transcription factor JunB by NEDD4L to enhance T_H_2 differentiation (84). The number of lung tumors in SGK1-deficient mice was much less than that in control mice after injection of melanoma cells into the tail vein (84). This finding indicated that NEDD4L might exert antitumor effects in melanoma. However, a study found that the expression of NEDD4L in cutaneous melanoma and lymph node metastatic melanoma was higher than that in normal melanocytes or benign nevus tissue (85). Overexpression of NEDD4L promoted the growth of A2058 melanoma cells *in vivo*, and downregulation of NEDD4L reduced the growth of G361 melanoma cells *in vitro* (85). Therefore, the role of NEDD4L in the development of melanoma is controversial and needs to be further clarified.

The expression of NEDD4L was in negatively correlated with the pathological grade of malignant glioma, and low expression of NEDD4L indicated poor outcomes (86). In addition, NEDD4L was reported as a direct target of miR-513a-5p, delayed the growth of glioma cells and amplified the cytotoxicity of temozolomide (TMZ) (87). Moreover, NEDD4L mediates the ubiquitination of the tumor oncogene sphingosine kinase 2 (SphK2) and thus suppresses the development of malignant glioma (88). Consequently, NEDD4L mainly crippled the malignant biological behavior of glioma. The role of NEDD4L in lymphoma has also been investigated. A study demonstrated that the expression of NEDD4L in Sezary syndrome (SS) was much higher than that in healthy controls (89). It was reported that NEDD4L might be involved in the progression of diffuse large B cell lymphoma (DLBCL) (90). Nevertheless, the specific effect of NEDD4L on lymphoma has not been confirmed. NEDD4L was found to be downregulated and correlated with biosynthesis and metabolism in clear-cell renal cell cancer (ccRCC) by integrated bioinformatics analysis. Low expression of NEDD4L was associated with dismal prognosis in ccRCC, suggesting that NEDD4L could act as a prognostic biomarker and therapeutic target in ccRCC (72). A bioinformatics analysis revealed that NEDD4L was downregulated in esophageal cancer patients and might be associated with esophageal cancer prognosis (91).

## Targeting NEDD4L for Cancer Therapy

Emerging evidence has demonstrated that several compounds can target the expression of NEDD4L. For example, β,β-dimethyl-acryl-alkannin (ALCAP2), a natural small-molecule compound separated from the root of Lithospermum erythrorhizon, could increase the expression of NEDD4L to hinder the nuclear translocation of β-catenin and stimulate the conjunction of ubiquitin and β-catenin, thus blocking the Wnt signaling pathway and inhibiting cell proliferation and metastasis as well as inducing apoptosis and cell cycle arrest in LUAD (92). Dexamethasone, a pretreatment drug, has been used for cancer treatment to reduce the toxic effect of chemotherapy. Dexamethasone was reported to promote the lung metastasis of breast cancer by regulating the PI3K-SGK1-CTGF pathway through the NEDD4L-Smad2 axis (93). AG490, an inhibitor of the Janus tyrosine kinase 2 (JAK2), can promote human organic anion transporter-3 (hOAT3) ubiquitination and degradation by enhancing the binding of NEDD4L with hOAT3 and reducing NEDD4L phosphorylation (94). Wogonin, a natural flavonoid agent, was revealed to upregulate the expression of NEDD4L and suppress the PI3K/Akt pathway (95). Therefore, targeting NEDD4L with these compounds might be helpful for cancer therapy in the future.

## Conclusions

**Figure 2:**
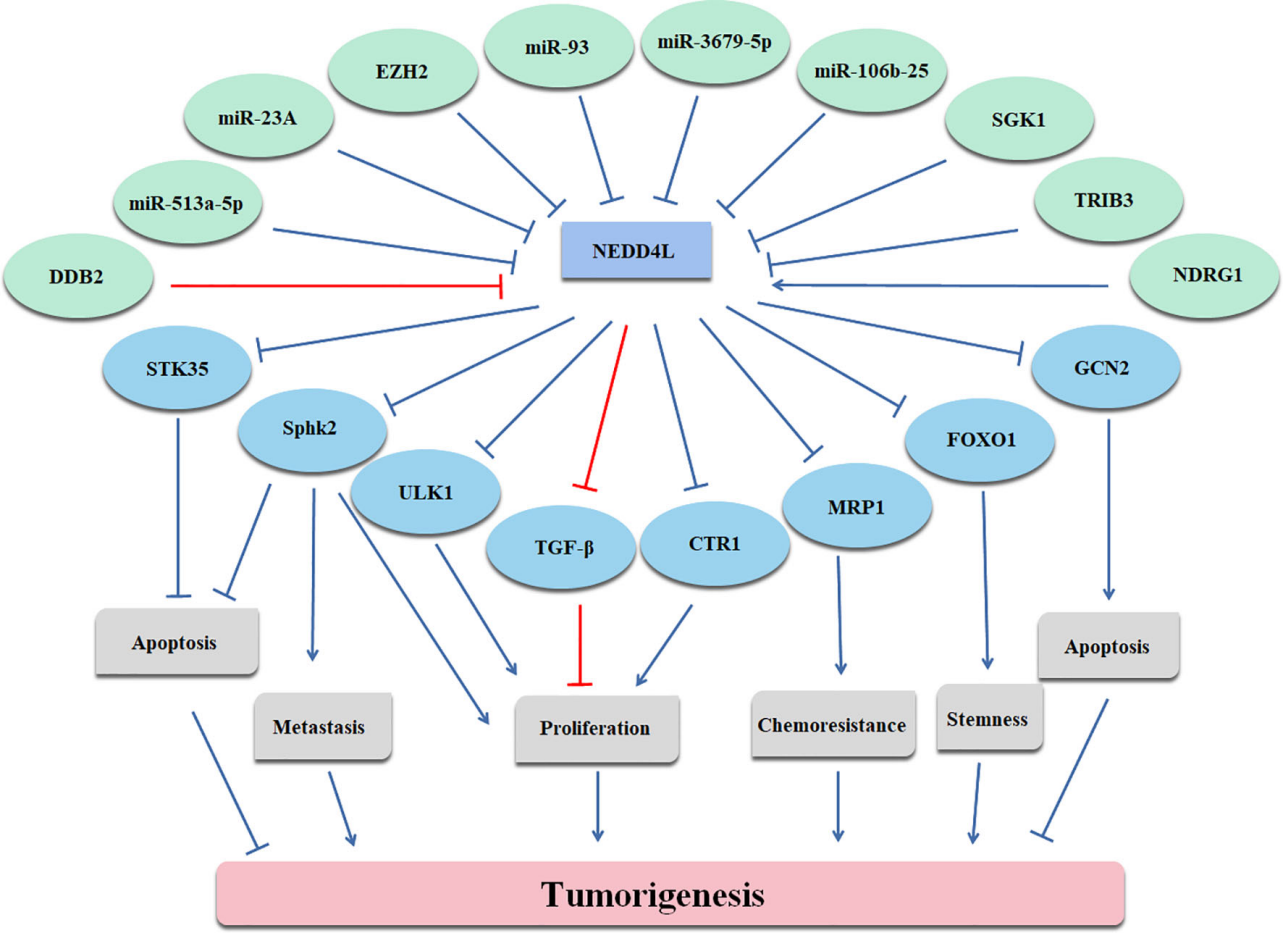
Upstream and downstream genes of NEDD4L are illustrated.

**Figure 3:**
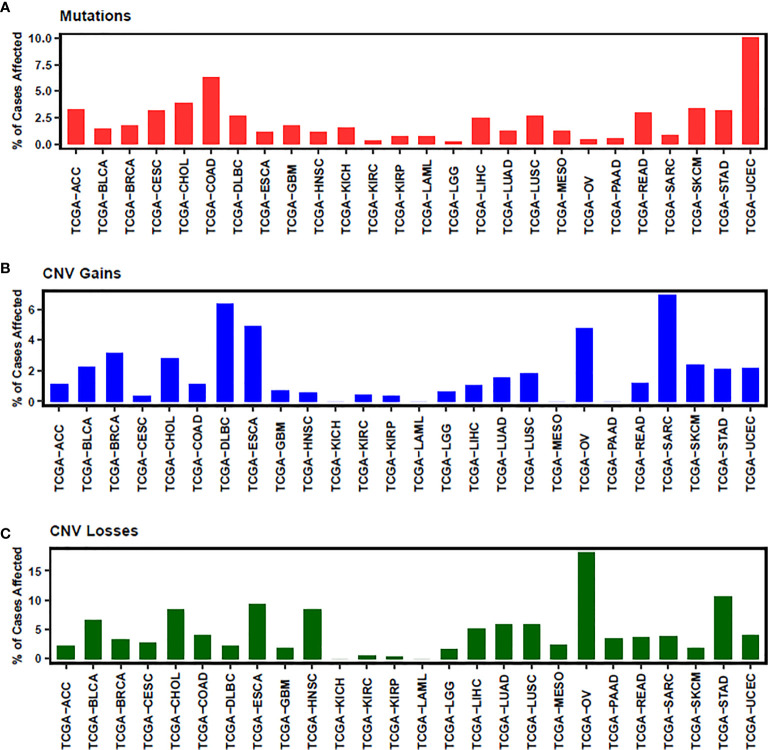
The status of mutation **(A)**, CNV gains **(B)** and CNV losses **(C)** of NEDD4L is illustrated. ACC, Adrenocortical carcinoma; BLCA, Bladder Urothelial Carcinoma; BRCA, Breast invasive carcinoma; CESC, Cervical squamous cell carcinoma and endocervical adenocarcinoma; CHOL, Cholangiocarcinoma; COAD, Colon adenocarcinoma; DLBC, Lymphoid Neoplasm Diffuse Large B-cell Lymphoma; ESCA, Esophageal carcinoma; GBM, Glioblastoma multiforme; HNSC, Head and Neck squamous cell carcinoma; KICH, Kidney Chromophobe; KIRC, Kidney renal clear cell carcinoma; KIRP, Kidney renal papillary cell carcinoma; LAML, Acute Myeloid Leukemia; LGG, Brain Lower Grade Glioma; LIHC, Liver hepatocellular carcinoma; LUAD, Lung adenocarcinoma; LUSC, Lung squamous cell carcinoma; MESO, Mesothelioma; OV, Ovarian serous cystadenocarcinoma; PAAD, Pancreatic adenocarcinoma; READ, Rectum adenocarcinoma; SARC, Sarcoma; SKCM, Skin Cutaneous Melanoma; STAD, Stomach adenocarcinoma; UCEC, Uterine Corpus Endometrial Carcinoma.

In conclusion, NEDD4L suppresses the carcinogenesis by elevating the degradation of substrates of NEDD4L in most cancer types, while the role of NEDD4L in a few cancer types still remains controversial (**Figure 2** and **Table 1**). Modulating upstream genes can affect the expression of NEDD4L and thus influence the progression of cancers (**Figure 2** and **Table 2**). It is important to mention that NEDD4L has mutations, copy number variations (CNV) gains, and CNV losses in a variety of human cancers (**Figure 3**). In some specific malignant tumors, the design of the NEDD4L enhancer may contribute to the better treatment of cancer patients, but the targeted inhibition of NEDD4L probably controls the procedure of some other cancer types. Additionally, the concrete mechanisms have not been fully elucidated, although there are abundant studies proving that NEDD4L plays oncogenic or tumor-suppressive roles in cancers by modulating its substrates. More detailed mechanistic research should be carried out to fully understand the function and role of NEDD4L in tumorigenesis. It is also critical to design and develop NEDD4L enhancers and inhibitors for cancer treatment in patients with dysregulation of NEDD4L. We believe that the clinical application of NEDD4L enhancers or inhibitors in cancer therapy will be prospective in the near future.

**Table 1:** NEDD4L gene modulates downstream genes and its impact on cancers.

Cancer	Downstream gene	Function	Reference
Colorectal cancer	STK35	accelerates glycolysis, decreases apoptosis, attenuates chemosensitivity	(42)
Melanoma	STK35	enhances TH2 differentiation	(84)
Pancreatic cancer	LTF	binds to iron and transports iron	(47)
	ULK1	involves in initiating cell autophagy	(30)
Lung cancer	MRP1	relates to multidrug resistance	(59)
	GCN2	increases the cell apoptosis	(60)
Breast cancer	NOTCH1	takes part in TIC induction	(75)
Ovarian cancer	TGF-β	inhibits the cell proliferation	(28)
	FOXO1	promotes breast cancer stemness	(77)
	CTR1	is the predominant transporter of copper and elevates the development of cancer	(78)
Glioma	SphK2	increases the cell proliferation and EMT	(88)

**Table 2:** Upstream gene modulates NEDD4L gene and its impact on cancers.

Cancer	Upstream gene	Function	Reference
Pancreatic cancer	miR-23A	promotes proliferation, migration and invasiveness	(45, 96)
	NDRG1	suppresses iron-regulated metastasis	(46)
Lung cancer	EZH2	regulates cell cycle, apoptosis and metastasis	(51)
	miR-93	enhances TGF-β-induced EMT	(57)
	miR-3679-5p	stabilizes the c-Myc protein to induce chemoresistance	(58)
Ovarian cancer	DDBP2	participates in gene transcription and cell cycle regulation	(65)
Breast cancer	miR-106b-25	takes part in TIC induction	(75)
	TRIB3	possesses tumor initiation capacity	(77)
Glioma	miR-513a-5p	influences the growth of glioma cells and reduces the cytotoxicity of TMZ	(87)

## Author Contributions

SX and XZ wrote this manuscript. LX and HL searched the literature regarding NEDD4L and cancers. YS and SX prepared the figures and tables. Z-wW was critically involved in discussion. All authors contributed to the article and approved the submitted version.

## Conflict of Interest

The authors declare that the research was conducted in the absence of any commercial or financial relationships that could be construed as a potential conflict of interest.

## Publisher’s Note

All claims expressed in this article are solely those of the authors and do not necessarily represent those of their affiliated organizations, or those of the publisher, the editors and the reviewers. Any product that may be evaluated in this article, or claim that may be made by its manufacturer, is not guaranteed or endorsed by the publisher.
